# 

**Published:** 1859-04

**Authors:** 


American Medical Association............................................ ....... ...

MISCELLANEOUS.

Chicago Academy of the Medical Sciences................... ............... .. .... 255

Statistics of Medical Colleges............................................................ 256

University of the Pacific........................................... .................... 257

Titles of Articles in Exchanges.......... ................................. ................... 258

Chicago Medical Society......................................................................... 260

### RECEIPTS FOR THE JOURNAL UP TO APRIL 15, 1859.

### ILLINOIS.

| | |
|---|---|
| Dr. J. P. Lynn, | vol. 2, $2 00 |
| " H. K. Lathy, | " 1, 2 00 |
| " F. W. Hance, | " 2, 2 00 |

### INDIANA.

| | |
|---|---|
| Drs. Latta & Jackson, | vol. 1, $2 00 |
| Dr. John F. Summers, | " 2, 2 00 |
| " Benj. F. Keith, | " 2, 2 00 |

### WISCONSIN.

| | |
|---|---|
| Dr. J. F. Hopkins, | vol. 2, $2 00 |

### OHIO.

| | |
|---|---|
| Dr. J. F. Spain, | vol. 2, $2 00 |

### CALIFORNIA.

| | |
|---|---|
| Dr. Ira E. Oatman, | vol. 2, $2 00 |

TITLES OF SOME OF THE PRINCIPAL ARTICLES IN OUR EXCHANGE JOURNALS.

*American Journal of the Medical Sciences.*—Carcinoma, *Woodward:* Carbunclar Inflammation of the Lip, *Lente:* Criminal Abortion, *Storer:* Encysted Tumor of Ovary, *Miller:* Effect of Alcohol, Glycerine, etc., on Exposed Hearts of Frogs, etc., *Mitchell:* Colorless Blood Corpuscle, *Hammond:* Gangrene of the Lungs, *Darrach:* Fracture at Base of Cranium, *Lockwood:* Extension Apparatus for Fractures, *Chapin:* Isthmus of Panama and Hospitals of Havana, *Horner.*

*North-American Medico-Chirurgical Review.*—Obstetric Jurisprudence (No. 2), *H. R. Storer:* Medicine in China, *J. J. Kerr:* Exsection of Sciatic Nerve for Neuralgia of Stump following Amputation of Thigh, *G. C. Blackman:* Proceedings of Pathological Society of Philadelphia.

*New York Journal of Medicine.*—Management of Shoulders in Examining Chest, *Corson:* Diseases Treated at New York Eye Infirmary, *Bumsted:* The Hymen, *Thomas:* The High Operation for Stone in the Bladder, *Hewit:* Respiratory Sounds, *Herzka:* Proceedings of the Medical Societies and of the Practice of New York Hospitals, *Shrady.*

*American Medical Monthly.*—The Elliptical Artificial Tympanum, *Clark:* Surgical Cases, *Briddon:* Medical Practitioners of Ancient Rome, *Peaslee.*

*Charleston Medical Journal.*—Cerebral Neuralgia terminating by Serous Apoplexy, *Da Costa:* Chancre of the Uterus, *Kollock:* Yellow Fever treated as an Exanthem, *Ogier:* Arnica-Montana, *Talley:* Treatment of Dysentery, *Forwood:* Pathology and Treatment of Yellow Fever, *Porcher.*

*Pacific Medical and Surgical Journal.*—Syphilis and its Treatment, *Toland:* Diphtheria, *Blake.*

*London Lancet.*—Diphtheria, *Ranking:* Strumous Ophthalmia, *Henry:* Lithotomy in Children, *Adams:* Ischyl, its Water and Baths, *Bennet:* Case of Hydrarthrosis of Knee-joint treated by Tapping and Iodine externally, *Hodge:* Case of Ununited Fracture, *Davies:* After-Pains, *Willis:* Syphilitic Inoculation, *Lee:* Report of *Lancet* Sanitary Committee on Diphtheria; Diphtheria, *Ramskill:* Sanitary Condition of the British Army.

*Montreal Medical Chronicle.*—Myeloid Tumor, *Howard:* Case of Twins with Single Placenta, *Grant:* Hour-glass Contraction of Uterus with the Fœtus, *McGarvin.*

*Nashville Journal of Medicine and Surgery.*—Graduating Address, *Singleton:* Pathological Geology, *Shapard:* Fevers, their Identity, etc., *Johnson.*

*Nashville Monthly Record.*—Clinical Lecture on Hernia, *May.*

*New Orleans Medical News and Hospital Gazette.*—Chemistry of Respiration, *Crawcour:* London Correspondence: Hospital Reports.

*St. Louis Medical and Surgical Journal.*—"Bruit de Pot Fele," *Gilfillan:* Stomatitis Materna, *Pallen:* Large Doses of Ipecacuanha in Dysentery, *McPheeters:* Retroversio Uteri, *Bolinger:* Proceedings of the St. Louis Medical Society.

*Oglethorpe Medical and Surgical Journal.*—Rational vs. Routine and Book Practice, *Smith:* General Dropsy, *Dozier:* Case of Stabbing in Abdomen, *Byrd:* Case of Fractured Cranium, *Mims.*

*Cincinnati Lancet and Observer.*—Bronzed Skin with Disease of Supra-Renal Capsules, *Krause:* Opium in Parturient Process, *Logan:* Fœtal Malformation, *Browning:* Laceration of Scrotum, *Bowman;* Injury to the Knee-Joint, *Haughton.*

*Ohio Medical and Surgical Journal.*—The Cole Case, *Mixer;* A case of Gun Shot Wound, *Culbertson.*

*Buffalo Medical Journal.*—Partial Dislocations, *Mercer;* Esquisse of Parisian Medicine and Surgery, *Mack*; Erysipelas, *Gay;* Prof. Hamilton's Clinics.

*Southern Medical and Surgical Journal.*—Malarial Fever, *Jones;* Case of Lithotomy, *Hammond;* Horse-shoe Pessary in Retroversion of Uterus, *W. B. Jones.*

*Virginia Medical Journal.*—Puerperal Phlebitis and Purulent Infection, *Thweatt;* Epidemics of Piedmont, *Payne;* Irregular Uterine Contractions, *Dashiell;* Traumatic Cerebritis, *Brock.*

*Atlanta Medical and Surgical Journal.*—Chloroform in Labor, *Logan;* Cases and Clinics.

*Louisville Medical Gazette* (semi-monthly).—Inguinal Hernia, *Goodwin;* Gun Shot Wounds, *Cook;* Sulphur as a Dentifrice, *Wright.*

*Semi-Monthly Medical News.*—Statistics of Hernia, *Richardson;* Case of Traumatic Tetanus successfully treated by Atropia, *Editors;* "Nature and Art," *Speed;* Medical Education.

*Peninsular and Independent.*—The Whitney Case: Case of Idiopathic Tetanus, *Fairbank;* Veratrum Viride, *Patterson.*

*Maine Medical and Surgical Reporter.*—A Plea for the Children.

*Medical and Surgical Reporter* (weekly).—Veratrum Viride, *Foster;* Fractures through the Inferior Extremity of the Radius, *Smith;* Kameela, the new Anthelmintic, *Boisnot;* Regional Anatomy, *Agnew;* Tuberculosis, *Ziegler;* Pancreatics, *Lawrence;* Amaurosis Treated by Strychnia, *Jaquett;* Full Reports from Philadelphia Hospitals.

*Boston Medical and Surgical Journal* (weekly).—Parasitical Disease of the Scalp; Treatment of certain Diseases of the Lachrymal Passages, *Williams.*

*American Medical Gazette.*—Uterine Speculum, *Woodward;* Veratrum Viride; Palmer's Patent Hand, Arm and Leg.

*Belmont Medical Journal.*—Can Quackery be Stopped? Veratrum Viride, *Estep.*

*The Scalpel.*—"An entirely original Quarterly Expositor of the Laws of Health and Abuses of Medicine and Domestic Life."

*American Journal of Pharmacy.*

*Journal of Materia Medica.*

*American Journal of Dental Science.*

*Dental News Letter.*

*Dental Register of the West.*

---

WANTED.

Medical Students, to act as Agents for the *Journal.*

---

CHICAGO MEDICAL SOCIETY.

This is the "Cook County Medical Society" christened with a new name. We are happy to be able to state that it is in a flourishing condition. At its last annual meeting, the following gentlemen were chosen officers for the current year:

*President,* Dr. D. D. Waite.
*Vice-President,* Dr. Swayne Wickersham.
*Secretary and Treasurer,* Dr. N. S. Davis.

*Delegates to the Americal Medical Association.*

Dr. N. S. Davis, Dr. Swayne Wickersham, and Dr. O'Ryan.

*Delegates to State Medical Society.*

Drs. Woodworth, Amerman, Holmes and Clapp.

---

It is suggested that, when doctors fight duels, the weapons used should be pills. We fear the suggestion will alarm non-professional readers.

